# Unveiling the dual threat: combined elevated triglyceride-glucose index and intracranial arterial stenosis burden for enhanced stroke risk stratification

**DOI:** 10.3389/fneur.2025.1561329

**Published:** 2025-06-30

**Authors:** Baoyi Liao, Guilin Meng, Xueyuan Liu

**Affiliations:** ^1^Department of Neurology, Shanghai Tenth People's Hospital, Tongji University School of Medicine, Shanghai, China; ^2^Tongji University School of Medicine, Shanghai, China; ^3^Department of Neurology, Tongren Hospital, Shanghai Jiao Tong University School of Medicine, Shanghai, China

**Keywords:** triglyceride glucose index, intracranial stenosis, atherosclerosis, ischemic stroke, stroke recurrence

## Abstract

**Background:**

The relationship between an elevated triglyceride-glucose index (TyG) combined with intracranial arterial stenosis (ICAS) and stroke recurrence remains incompletely understood. This study investigated how varying ICAS burdens influence the prognosis of ischemic stroke patients with different TyG levels.

**Methods:**

This prospective cohort study analyzed a consecutively enrolled population of 712 acute ischemic stroke patients, with intracranial atherosclerotic burden quantitatively evaluated via computed tomography angiography (CTA) or three-dimensional time-of-flight magnetic resonance angiography (MRA), and stenosis severity graded according to WASID criteria. Participants were categorized into four mutually exclusive subgroups based on TyG index quartiles and ICAS status (presence/absence). To investigate exposure-response gradients, further stratification incorporated both ICAS severity (no/mild [<50%], moderate [50–69%], or severe [70–99% or occlusion] stenosis) and vascular involvement (single vs. multiple affected arterial territories). The primary endpoint was defined as ischemic stroke recurrence within a standardized 90-day follow-up period. Cox proportional hazards models examined adjusted associations between TyG/ICAS status and outcomes, complemented by Kaplan-Meier curves with log-rank tests for time-to-event visualization. Mechanistic pathways were delineated through causal mediation analysis employing Quasi-Bayesian approximation (100 Monte Carlo simulations) to quantify mediating effects between metabolic dysfunction and cerebrovascular outcomes.

**Conclusion:**

The synergistic combination of elevated TyG index and ICAS burden demonstrated superior prognostic value for 3-month stroke recurrence risk compared to either parameter independently, suggesting that concurrent assessment of insulin resistance (via TyG index) and vascular imaging biomarkers (via ICAS burden) may refine early risk stratification. This finding underscores the importance of integrating systemic metabolic dysfunction with structural cerebrovascular pathology to identify patients at heightened vulnerability during the critical post-stroke recovery window.

## Introduction

Insulin resistance (IR) is an independent risk factor for atherosclerosis, coronary heart disease and stroke, affecting a significant proportion of the general population (1–3). A study of 72 non-diabetic patients with ischemic stroke found that 50% had significant IR (4). Meta-analysis data showed a 2.55-fold increased risk of stroke in insulin-resistant populations compared with the general population after adjustment for conventional confounders (5). Similarly, population-based cohort studies have identified IR as a marker of increased stroke risk in non-diabetic individuals (6).

At present, IR is conspicuously absent from post-stroke risk stratification protocols. The relationship between type 2 diabetes mellitus (T2DM) and atherosclerotic plaque formation goes beyond simple hyperglycaemia. IR is a distinct risk factor for atherosclerosis, exerting several proatherogenic effects even in the absence of T2DM (7–9). This metabolic dysfunction may precede T2DM diagnosis by years or decades, with atherosclerotic processes potentially initiating long before diabetes becomes clinically apparent (10). While the hyperinsulinemic normoglycemic clamp test (HOMA-IR) remains the gold standard for measuring insulin resistance, its complexity and resource-intensive nature limit its utility in routine clinical practice and large-scale epidemiological research (11). The TyG index has emerged as a practical, cost-effective, and intuitive measure of insulin resistance, with substantial epidemiological evidence supporting its association with insulin resistance (12–14).

ICAS occurs frequently in Chinese populations, with previous studies demonstrating that increased ICAS burden correlates strongly with stroke recurrence, more severe neurological deficits, and poor clinical outcomes (15–19). Current clinical practice lacks definitive pharmacological or endovascular interventions to eliminate stroke recurrence risk in ICAS patients. Consequently, identifying those at highest risk for recurrence among the ICAS population remains clinically imperative.

Systemic metabolic disturbances, as reflected by the TyG index, and local haemodynamic disturbances, as reflected by ICAS, may have a dual synergistic effect: the former exacerbates blood–brain barrier disruption through a proinflammatory state, and the latter promotes expansion of the ischemic hemidarkness zone through hypoperfusion, which together accelerate the process of neuronal apoptosis. The ability of the TyG index in combination with ICAS to serve as a highly efficient and cost-effective risk stratification tool to accurately screen patients with a poor prognosis in stroke needs to be further evaluated in prospective cohorts. Patients with poor prognosis need further evaluation in prospective cohorts. Based on current clinical needs, the aim of this study was to investigate the synergistic effect of the TyG index combined with ICAS on short-term recurrence of ischemic stroke.

## Methods

### Ethics statement

This observational cohort study was approved by the Ethics Committee of Shanghai Tenth People’s Hospital and conducted in accordance with the Declaration of Helsinki guidelines.

### Study population

The study cohort comprised patients diagnosed with acute ischemic stroke who underwent CTA or MRA evaluation at Shanghai Tenth People’s Hospital between August 2023 and August 2024. Ischemic stroke diagnosis was established through assessment of clinical manifestations and neuroimaging findings from brain CT or MRI scans. The final analysis included 712 patients. The X-tile software was employed to determine the optimal TyG threshold for distinguishing elevated stroke recurrence risk, which formed the basis for stratifying patients into lower and higher TyG groups.

Exclusion criteria encompassed: transient ischemic attack (TIA); treatment with recombinant tissue plasminogen activator (rt-PA) or endovascular intervention; presence of infections, neoplasms, hematological disorders, autoimmune conditions, chronic liver disease, or renal disease; and modified Rankin score (mRS) greater than 2.

### Image acquisition

Cerebral vascular stenosis severity and distribution were evaluated using CTA or MRA imaging. Vascular assessment followed the Warfarin and Aspirin for Symptomatic Intracranial Arterial Stenosis (WASID) methodology (20). ICAS was defined as atherosclerotic stenosis or occlusion exceeding 50% in any major intracranial artery. The evaluated vessels included: middle cerebral artery (M1–M2 segments), anterior cerebral artery (A1–A2 segments), posterior cerebral artery (P1–P2 segments), vertebral artery (V4 segment), internal carotid artery (C6–C7 segments), and basilar artery.

ICAS severity was categorized as: no/mild stenosis (<50%), moderate stenosis (50–69%), or severe stenosis (70–99% or occlusion). ICAS burden was classified according to the number of affected vessels: no stenosis, single vessel involvement, or multiple vessel involvement.

### Clinical data collection

Patient information was obtained from computerized medical records. Baseline characteristics included age, gender, body mass index (BMI), current smoking and alcohol consumption status, and admission systolic and diastolic blood pressures (SBP and DBP). Medical history documentation encompassed hypertension, diabetes, atrial fibrillation, previous stroke, and coronary atherosclerotic heart disease.

Laboratory assessments included total cholesterol (TC), triglycerides (TG), high-density lipoprotein cholesterol (HDL-C), low-density lipoprotein cholesterol (LDL-C), and fasting blood glucose (FBG). Blood samples were collected after a minimum 8-h fasting period and analyzed within 24 h of admission. Stroke severity was assessed by qualified neurologists using the National Institutes of Health Stroke Scale (NIHSS). The TyG index was calculated as Ln[TG(mg/dL) × FBG(mg/dL)/2] (21).

### Outcome assessment

Stroke recurrence was defined as an acute new focal neurological deficit lasting more than 24 h with an increase in NIHSS score of 4 or more; or on the basis of imaging evidence (new infarct or enlargement of the original infarct area) on MRI or CT scan (22).

### Statistical analysis

Continuous variables were expressed as means with standard deviations and analyzed using t-tests, while categorical variables were presented as counts with percentages and evaluated using chi-square tests.

Cox regression models were employed to analyze the relationships between TyG index, ICAS status, and ischemic stroke recurrence. Two analytical models were constructed: Model 1 presented unadjusted analyses, while Model 2 incorporated adjustments for demographic characteristics (gender, age, SBP, DBP, current smoking status, current alcohol consumption), medical history (hypertension, diabetes, stroke, atrial fibrillation, coronary heart disease), hospitalization treatment (antiplatelet therapy, statin therapy), and laboratory parameters (HDL and LDL levels). Multicollinearity testing confirmed the absence of significant correlation among adjusted variables, with variance inflation factors and tolerance values presented in Appendix.

The cumulative risk of stroke recurrence over the 90-day follow-up period was visualized using Kaplan–Meier curves. To assess the incremental predictive value of combining TyG index and ICAS status beyond conventional risk factors, we calculated the C-statistic, integrated discrimination improvement (IDI), and net reclassification index (NRI). Additionally, causal mediation analysis evaluated the magnitude of potential mediating effects between variables. All statistical tests were two-tailed, with *p*-values less than 0.05 considered statistically significant.

## Results

### Baseline characteristics

The baseline characteristics of the enrolled patients are shown in Table 1. Of the 712 patients enrolled, 353 (49.57%) were male. Comparative analysis showed that patients in the higher TyG index group were significantly younger (66 vs. 69 years, *p* < 0.001) and had higher BMI, SBP and DBP. Laboratory analyses showed increased levels of TG, glucose, TyG index, TC, HDL and LDL in this group (all *p* < 0.01). The lower TyG group had a lower prevalence of hypertension and diabetes. However, this group had a higher incidence of atrial fibrillation (all *p* < 0.01). Cardiogenic embolism was more common than atherosclerotic stroke in the lower TyG group, which also had lower proportions of severe stenosis and multiple vessel involvement.

**Table 1:** Baseline characteristics based on TyG index and ICAS status.

Characteristic	Lower TyG index (*n* = 360)	Higher TyG index (*n* = 352)	*p*-value	Without ICAS (*n* = 305)	With ICAS (*n* = 407)	*p*-value
Demographic characteristics						
Male sex, *n* (%)	172 (47.8%)	181 (48.6%)	0.261	156 (51.1%)	197 (48.4%)	0.469
Age, year, mean (SD)	69 ± 10	66 ± 10	<0.001	66 ± 10	68 ± 10	0.020
BMI, kg/m^2^, mean (SD)	23.9 ± 3.4	24.9 ± 3.4	<0.001	24.3 ± 3.5	24.5 ± 3.3	0.495
Baseline SBP, mmHg, mean (SD)	146 ± 19	152 ± 21	<0.001	148 ± 20	149 ± 21	0.344
Baseline DBP, mmHg, mean (SD)	86 ± 13	89 ± 13	0.006	89 ± 13	87 ± 13	0.025
Current cigarette smoking, *n* (%)	106 (29.4%)	95 (27.0%)	0.560	86 (28.2%)	115 (28.3%)	0.986
Current alcohol drinking, *n* (%)	34 (9.4%)	39 (11.1%)	0.387	32 (10.5%)	41 (10.1%)	0.856
Admission NIHSS score Median (IQR)	2 (1, 4)	3 (1, 4)	0.510	3 (1, 4)	2 (1, 4)	0.592
Clinical features, mean (SD)						
Triglyceride, mmol/L	0.99 ± 0.28	1.88 ± 0.81	<0.001	1.33 ± 0.65	1.51 ± 0.81	<0.001
Plasma glucose, mmol/L	4.96 ± 0.91	7.50 ± 2.90	<0.001	5.75 ± 1.97	6.56 ± 2.76	<0.001
TyG index	8.22 ± 0.31	9.19 ± 0.45	<0.001	8.57 ± 0.57	8.79 ± 0.64	<0.001
Total cholesterol, mmol/L	4.13 ± 1.04	4.54 ± 1.07	<0.001	4.30 ± 1.01	4.35 ± 1.12	0.504
HDL cholesterol, mmol/L	1.14 ± 0.28	1.02 ± 0.23	<0.001	1.13 ± 0.28	1.05 ± 0.24	<0.001
LDL cholesterol, mmol/L	2.60 ± 0.91	2.82 ± 0.92	<0.001	2.67 ± 0.87	2.74 ± 0.96	0.370
Medical history, *n* (%)						
Hypertension	249 (69.2%)	280 (79.5%)	0.002	208 (68.2%)	321 (78.9%)	0.001
Diabetes	111 (30.8%)	239 (67.9%)	<0.001	131 (43.0%)	219 (53.8%)	0.004
Stroke	76 (21.1%)	86 (24.4%)	0.291	53 (17.4%)	109 (26.8%)	0.003
Atrial fibrillation	40 (11.1%)	21 (6.0%)	0.014	27 (8.9%)	34 (8.4%)	0.814
Coronary heart disease	57 (15.8%)	56 (15.9%)	0.978	41 (13.4%)	72 (17.7%)	0.125
Hospitalization treatment, *n* (%)						
Antiplatelet therapy			0.693			0.993
Single antiplatelet therapy	139 (38.6%)	141 (40.1%)		120 (39.3%)	160 (39.3%)	
Dual antiplatelet therapy	221 (61.4%)	211 (59.9%)		185 (60.7%)	247 (60.7%)	
Statin therapy	350 (97.2%)	339 (96.3%)	0.490	294 (96.4%)	395 (97.1%)	0.623
Stroke subtype, *n* (%)			<0.001			<0.001
Lacunar	166 (46.1%)	154 (43.7%)		199 (65.2%)	121 (29.7%)	
Large-artery atherosclerosis	114 (31.7%)	158 (44.9%)		31 (10.2%)	241 (59.2%)	
Cardioembolism	36 (10.0%)	20 (5.7%)		32 (10.5%)	24 (5.9%)	
Other	44 (12.2%)	20 (5.7%)		43 (14.1%)	21 (5.2%)	
ICAS, *n* (%)			<0.001	–	–	–
No	183 (50.8%)	122 (34.7%)		–	–	
Yes	177 (49.2%)	230 (65.3%)		–	–	
Multi-stenosis of ICAS, *n* (%)			<0.001			<0.001
No	276 (76.7%)	227 (64.5%)		305 (100.0%)	198 (48.6%)	
Yes	84 (23.3%)	125 (35.5%)		0 (0.0%)	209 (51.4%)	
Severe ICAS, *n* (%)			<0.001			<0.001
No	267 (74.2%)	214 (60.8%)		305 (100.0%)	176 (43.2%)	
Yes	93 (25.8%)	138 (39.2%)		0 (0.0%)	231 (56.8%)	

Data are presented as mean ± SD for continuous variables, median (interquartile range) for NIHSS scores, and *n* (%) for categorical variables. TyG, triglyceride-glucose; BMI, body mass index; SBP, systolic blood pressure; DBP, diastolic blood pressure; NIHSS, National Institutes of Health Stroke Scale; HDL, high-density lipoprotein; LDL, low-density lipoprotein; ICAS, intracranial arterial stenosis.

On stratification by ICAS status, 56.8% of the ICAS group had severe stenosis and 51.4% multiple vessel involvement. ICAS patients were slightly older (68 ± 10 vs. 66 ± 10 years, *p* = 0.020). Other variables such as BMI, SBP, DBP, current smoking status, alcohol consumption and admission NIHSS scores showed no significant differences between the groups. However, ICAS patients had higher levels of TG, glucose, TyG index, HDL and LDL and an increased prevalence of hypertension, diabetes and previous stroke (all *p* < 0.01).

### TyG index and stroke recurrence in different subgroups

Table 2 compares the associations between the TyG index and the risk of stroke recurrence in three subgroups: the total population (*n* = 712), the ICAS subgroup (*n* = 407), and the non-ICAS subgroup (*n* = 305). After multivariable adjustment, the TyG index as a continuous variable showed no significant association in the overall population (OR = 1.18, 95% CI: 0.72–1.95, *p* = 0.505); however, patients in the highest TyG quartile (Q4) had a 2.51-fold increased risk of recurrence compared to Q1 (OR = 2.51, 95% CI: 1.02–6.17, *p* = 0.045). In the ICAS subgroup, neither the continuous TyG index (OR = 1.28, 95% CI: 0.72–2.28, *p* = 0.400) nor the quartile-based analysis (Q4 vs. Q1: OR = 1.79, 95% CI: 0.60–5.29, *p* = 0.293) showed statistically significant associations with stroke recurrence risk. In the non-ICAS population, no significant differences in the risk of stroke recurrence were observed between subgroups.

**Table 2:** TyG index and stroke recurrence in different subgroups.

Characteristic	Model 1					Model 2		
	N	Event N	HR	95% CI	*p*-value	HR	95% CI	*p*-value
Association between the TyG index and risk of stroke recurrence: overall population								
TyG (continuous)	712	53	1.26	0.83, 1.91	0.284	1.18	0.72, 1.95	0.505
TyG (categories)								
TyG quartile 1	178	7	—	—		—	—	
TyG quartile 2	178	13	1.90	0.76, 4.76	0.172	1.95	0.77, 4.96	0.160
TyG quartile 3	178	13	1.89	0.75, 4.74	0.174	1.85	0.67, 5.07	0.232
TyG quartile 4	178	20	2.98	1.26, 7.06	0.013	2.51	1.02, 6.17	0.045
*p* for trend					0.114			0.205
Relationship between TyG index and risk of stroke recurrence: ICAS population								
TyG (continuous)	407	40	1.41	0.89, 2.23	0.147	1.28	0.72, 2.28	0.400
TyG (categories)								
TyG quartile 1	102	7	—	—		—	—	
TyG quartile 2	101	9	1.32	0.49, 3.53	0.586	1.23	0.43, 3.51	0.702
TyG quartile 3	102	10	1.45	0.55, 3.82	0.447	1.26	0.45, 3.52	0.662
TyG quartile 4	102	14	2.05	0.83, 5.08	0.121	1.79	0.60, 5.29	0.293
*p* for trend					0.112			0.301
Relationship between TyG index and stroke recurrence risk: non-ICAS population								
TyG (continuous)	305	13	1.24	0.49, 3.13	0.654	1.53	0.41, 5.77	0.529
TyG (categories)								
TyG quartile 1	76	3	—	—		—	—	
TyG quartile 2	76	3	0.99	0.20, 4.90	0.989	0.83	0.15, 4.61	0.829
TyG quartile 3	76	4	1.33	0.30, 5.95	0.707	0.91	0.16, 5.12	0.916
TyG quartile 4	77	3	0.98	0.20, 4.88	0.985	0.91	0.12, 6.95	0.931
*p* for trend					0.913			0.960

HR, Hazard Ratio; CI, Confidence Interval.

Model 1: No covariates were adjusted.

Model 2: Adjusted for age, sex, BMI, SBP, DBP, current cigarette smoking, current alcohol drinking, hypertension, diabetes, atrial fibrillation, coronary heart disease, stroke, antiplatelet therapy, statin therapy, HDL, LDL, and admission NIHSS score.

### Risk of stroke at 3 months by the status of TyG index and ICAS

During follow-up, 27 patients were lost to follow-up and 53 experienced recurrent ischemic stroke. Using patients with lower TyG levels and without ICAS as the reference group, those with elevated TyG index or ICAS demonstrated increased risk of 90-day stroke recurrence (Table 3). In the unadjusted model, patients with both higher TyG index and increased ICAS burden showed a 3.61-fold elevated recurrence risk (95% CI, 1.49–8.78; *p* = 0.005). This association persisted in the fully adjusted model, with a 3.21-fold increased risk (95% CI, 1.23–8.42; *p* = 0.018) in this group.

**Table 3:** Effect of TyG and ICAS burden on stroke recurrence.

Characteristic	Model 1				Model 2			
	N	Event N	HR	95% CI	*p*-value	HR	95% CI	*p*-value
Risk of stroke at 3 months by the status of TyG index and ICAS								
Lower TyG without ICAS	183	6	—	—		—	—	
Higher TyG without ICAS	122	7	1.78	0.60, 5.31	0.298	1.66	0.53, 5.25	0.388
Lower TyG with ICAS	177	14	2.48	0.95, 6.44	0.063	2.46	0.93, 6.49	0.069
Higher TyG with ICAS	230	26	3.61	1.49, 8.78	0.005	3.21	1.23, 8.42	0.018
*p* for trend					0.002			0.008
Risk of stroke at 3 months by the status of TyG index and number of ICAS segments								
Lower TyG without ICAS	183	6	—	—		—	—	
Higher TyG without ICAS	122	7	1.78	0.60,5.31	0.298	1.63	0.52,5.15	0.402
Lower TyG + 1 ICAS segment	93	6	2.01	0.65, 6.22	0.228	1.84	0.58,5.83	0.297
Higher TyG + 1 ICAS segment	105	8	2.37	0.82, 6.84	0.110	1.99	0.64,6.17	0.234
Lower TyG + multiple ICAS segment	84	8	3.01	1.04, 8.66	0.042	3.18	1.08,9.33	0.035
Higher TyG + multiple ICAS segment	125	18	4.71	1.87,11.87	0.001	4.24	1.57,11.45	0.004
*p* for trend					<0.001			<0.001
Risk of stroke at 3 months by the status of TyG index and ICAS severity								
Lower TyG without ICAS	183	6	—	—		—	—	
Higher TyG without ICAS	122	7	1.78	0.60, 5.31	0.298	1.66	0.52, 5.25	0.389
Lower TyG + moderate stenosis	84	5	1.84	0.56, 6.02	0.315	1.69	0.51, 5.65	0.392
Higher TyG + moderate stenosis	92	8	2.73	0.95, 7.87	0.063	2.38	0.77, 7.39	0.134
Lower TyG + severe stenosis	93	9	3.07	1.09, 8.62	0.033	3.32	1.15, 9.58	0.026
Higher TyG + severe stenosis	138	18	4.22	1.67,10.63	0.002	3.81	1.40,10.37	0.009
*p* for trend					<0.001			0.002

HR, Hazard Ratio; CI, Confidence Interval.

Model 1: No covariates were adjusted.

Model 2: Adjusted for age, sex, BMI, SBP, DBP, current cigarette smoking, current alcohol drinking, hypertension, diabetes, atrial fibrillation, coronary heart disease, stroke, antiplatelet therapy, statin therapy, HDL, LDL, and admission NIHSS score.

**Figure 1:**
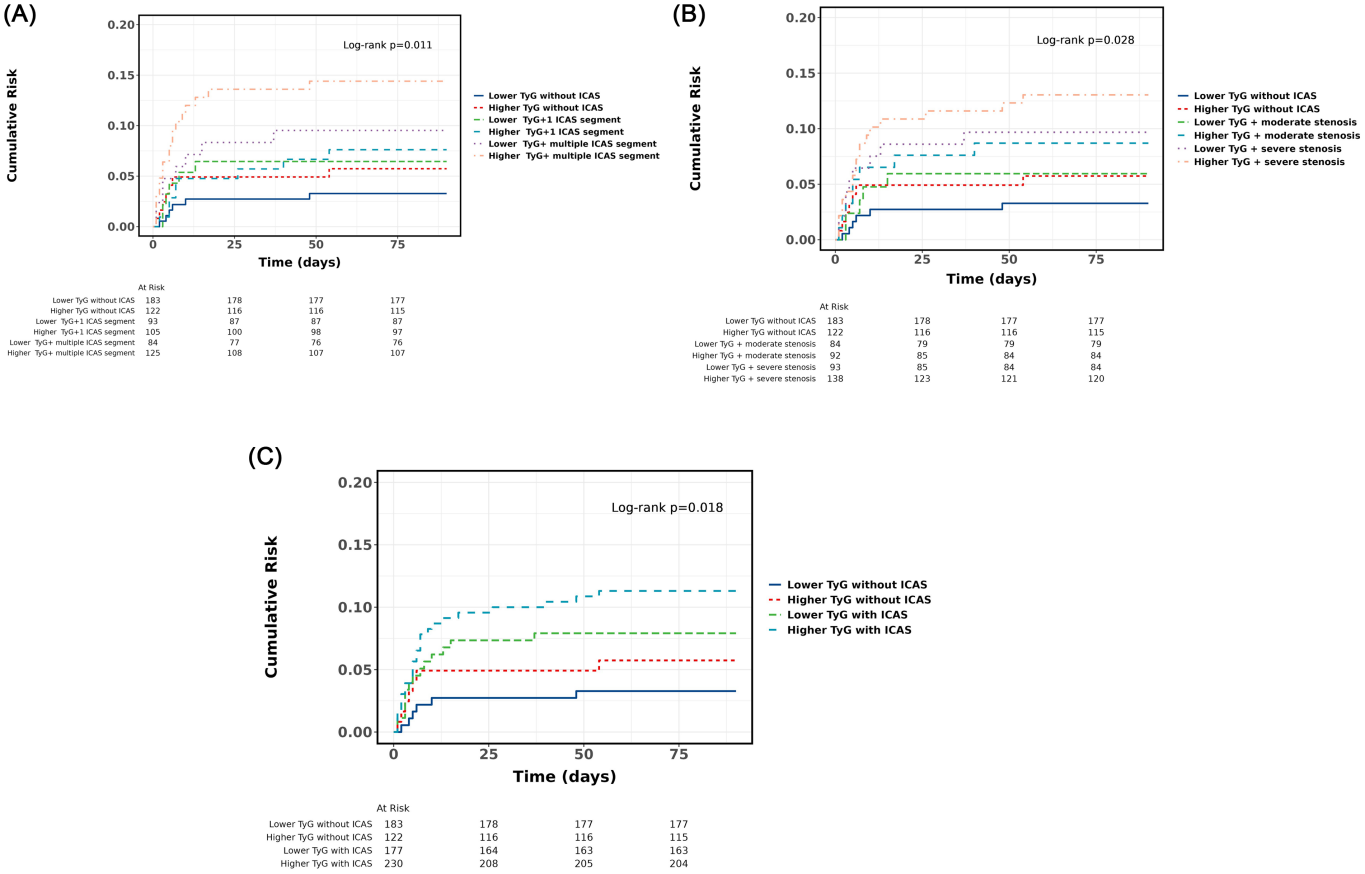
Cumulative risk of stroke recurrence over 90 days. **(A)** Kaplan-Meier survival curves of the TyG index in combination with ICAS subgroups. **(B)** Kaplan-Meier survival curves of the TyG index in combination with ICAS segments. **(C)** Kaplan-Meier survival curves of the TyG index in combination with ICAS stenosis subgroups.

Analysis revealed a dose-dependent relationship between ICAS severity and new stroke risk (*p* for trend <0.001). Compared to the reference group, patients with severe ICAS demonstrated increased stroke risk regardless of TyG status, though the risk was higher in the elevated TyG group (adjusted hazard ratio 3.81, 95% CI 1.40–10.37) than in the lower TyG group (adjusted hazard ratio 3.32, 95% CI 1.15–9.58). Similar patterns emerged when patients were stratified by number of affected ICAS segments, as illustrated in the Kaplan–Meier curves (Figure 1).

### The value of TyG + ICAS improved the risk stratification of stroke recurrence

The addition of TyG index and ICAS status significantly improved the predictive capability of the conventional risk model, with the C-statistic increasing from 0.689 to 0.714 (*p* = 0.045). Furthermore, this combined approach demonstrated enhanced discrimination and reclassification ability (IDI: 1.60%, *p* = 0.024; Continuous-NRI: 17.60%, *p* = 0.036) (Table 4).

**Table 4:** Improved risk stratification with combined TyG and ICAS assessment.

Variable		Estimate	95% CI	*p*-value
Conventional model	C-statistic	0.689	(0.614–0.764)	0.045
Conventional model + TyG and ICAS		0.714	(0.640–0.788)	
Conventional model	IDI	Reference	Reference	0.024
Conventional model + TyG and ICAS		1.6 (0.1–6.9)	(0.10–6.91)	
Conventional model	Continuous-NRI	Reference	Reference	0.036
Conventional model + TyG and ICAS		17.60	(1.80–31.20)	

TyG, triglyceride-glucose; ICAS, intracranial arterial stenosis; CI, confidence interval; IDI, integrated discrimination improvement; NRI, net reclassification index.

The conventional model included age, sex, BMI, SBP, DBP, current cigarette smoking, current alcohol drinking, hypertension, diabetes, atrial fibrillation, coronary heart disease, stroke, antiplatelet therapy, statin therapy, HDL, LDL, and admission NIHSS score.

### Causal mediation analysis

The total effect of ICAS on stroke recurrence showed statistical significance (coefficient 0.04827, 95% CI 0.00545–0.08880, *p* = 0.020). However, the indirect effect mediated through TyG demonstrated no statistical significance (coefficient −0.00126, 95% CI −0.00612 to 0.00271, *p* = 0.560). The direct effect of ICAS on stroke recurrence maintained significance (coefficient 0.04952, 95% CI 0.00590–0.09034, *p* = 0.020). The proportion mediated by TyG was estimated at −2.2% (95% CI −23.5, 7.4), indicating minimal mediation effect (Table 5).

**Table 5:** Mediation analysis for the associations between ICAS and stroke recurrence.

Independent variable	Mediator	Total effect		Indirect effect		Direct effect		Proportion mediated (95% CI)
		Coefficient (95% CI)	*p* value	Coefficient (95% CI)	*p* value	Coefficient (95% CI)	*p* value	
ICAS	TyG	0.04827 (0.00545, 0.08880)	0.020	−0.00126 (−0.00612, 0.00271)	0.560	0.04952 (0.00590, 0.09034)	0.020	−2.2 (−23.5, 7.4)

The mediation analyses were adjusted for age, sex, BMI, SBP, DBP, current cigarette smoking, current alcohol drinking, hypertension, diabetes, atrial fibrillation, coronary heart disease, stroke, antiplatelet therapy, statin therapy, HDL, LDL, and admission NIHSS score.

ICAS, intracranial arterial stenosis; TyG, triglyceride-glucose index; CI, confidence interval.

## Discussion

This study showed that patients with both an elevated TyG index and an increased ICAS burden had a significantly higher risk of recurrent ischemic stroke at 90 days. Our results showed a gradient relationship when stratified by stenosis severity and number of ICAS segments. Specifically, patients with an elevated TyG index and multiple ICAS stenoses had a 4.24-fold increased risk of recurrent stroke. Similarly, patients with severe stenosis had a 3.81-fold increased risk of short-term recurrence. This synergistic effect proved more predictive of new stroke risk than either elevated TyG levels or ICAS burden alone.

Several studies have validated the TyG index as a reliable indicator of insulin resistance (12, 23). The PURE study found that the TyG index independently predicted future cardiovascular mortality, myocardial infarction, stroke and T2DM, suggesting a fundamental role for IR in the pathogenesis of cardiovascular and metabolic diseases (14). Given the established diagnostic value of the TyG index for IR and these documented associations, we hypothesized a strong relationship between the TyG index and cerebrovascular disease.

There is increasing evidence that an elevated TyG index significantly influences vascular events and prognostic deterioration in ischemic stroke patients. In a multicentre study of 3,216 stroke patients in 22 Chinese hospitals, Miao M et al. found that increased TyG index correlated with higher rates of unfavorable functional outcome at discharge and in-hospital mortality, suggesting that TyG index significantly influences pathophysiological progression during the acute phase of ischemic stroke (24). The TyG index has also been shown to be associated with major adverse cardiac and cerebrovascular events, even in patients who have undergone endovascular intervention (25, 26).

Previous research has linked IR to platelet activation (27, 28). Basili S demonstrated the role of IR as a key determinant of platelet activation in obese women (29). Guo Y et al. showed that patients with acute ischemic stroke receiving dual antiplatelet therapy had increased platelet reactivity and a higher prevalence of aspirin-induced platelet reactivity when TyG index was elevated (30), which partially explains why patients receiving intensive antiplatelet therapy but with IR have a higher risk of stroke recurrence. These findings suggest that personalized therapeutic strategies to control the TyG index may offer viable approaches for secondary stroke prevention.

An elevated TyG index has been shown to facilitate both the initiation and progression of atherosclerosis, with established associations between TyG and carotid atherosclerosis (31) and plaque instability (32). Similar correlations have been demonstrated between elevated TyG levels and the severity of coronary artery stenosis (33, 34). As ICAS is an independent risk factor for stroke recurrence and has a particularly high prevalence in Asian populations (35, 36), the simultaneous presence of ICAS and an elevated TyG index may have a synergistic effect on the prediction of stroke recurrence.

This synergistic effect likely stems from multiple mechanisms. In insulin-resistant states, the anti-inflammatory and antiatherogenic benefits normally mediated by endothelial nitric oxide generation through the phosphatidylinositol-3-kinase pathway become attenuated (37–39). Simultaneously, the mitogen-activated protein kinase pathway in endothelial and vascular smooth muscle cells, along with its various proatherogenic effects, may be enhanced (40–43). This pathophysiological imbalance potentially mediates stenosis progression during the acute phase. Additionally, lesional macrophage apoptosis combined with impaired efferocytosis may result in increased plaque necrosis, consequently elevating thrombosis risk in pre-existing narrow arteries (44).

Furthermore, Hoscheidt SM et al. showed that increased IR levels correlated with decreased cerebral perfusion and arterial blood flow (45), and a Korean study showed that in acute ischemic stroke patients with middle cerebral artery or internal carotid artery occlusion, increased TyG index independently predicted poor outcome, even after reperfusion therapy (46). We propose that a higher TyG index in patients with large vessel occlusion may indicate reduced perfusion levels, contributing to suboptimal neurological recovery.

Our study also shows that people with a higher ICAS burden have the highest risk of stroke recurrence. Elevated ICAS levels indicate extensive vasculopathy and multiple vascular risk factors, which contribute to a significantly higher risk of recurrent infarction in the acute phase. Therefore, patients with higher ICAS burden combined with elevated TyG warrant more intensive follow-up and aggressive secondary prevention due to their increased risk of recurrence.

This investigation revealed significant direct effects of ICAS on stroke recurrence but did not support substantial mediation through TyG. The direct pathway suggests alternative mechanisms through which ICAS influences recurrent stroke, independent of metabolic factors captured by TyG. The negative proportion mediated further indicates that TyG plays a minimal role in this causal pathway, warranting investigation of other potential mediators.

## Study limitations

This study has several important limitations. First, digital subtraction angiography (DSA) is not routinely used as an intracranial vascular screening tool at our center. Although MRA/CTA shows high agreement with DSA, the most accurate vascular assessment is not yet available. However, non-invasive and readily available screening tools may offer broader clinical applicability than DSA for routine patient assessment.

Second, defining this heterogeneous disease solely based on lumen size potentially overlooks other high-risk markers, including non-stenotic but high-risk carotid plaque (47), vessel calcification (48) inflammatory status (49), and cerebral small vessel disease burden scores (50). Future implementation of higher spatial resolution techniques to detect plaque composition, vessel wall changes and the dynamic evolution of the collateral circulation would improve the differentiation of atherosclerotic progression. Given potential synergistic effects, the integration of multiple biomarkers could optimize the identification of truly high-risk patients. Third, stratification by intracranial vascular burden necessarily limited our subgroup sample sizes, potentially reducing statistical power. Larger analyses are needed to validate the generalisability of our findings. Fourth, we did not serially monitor changes in the TyG index over time, which may have limited our ability to detect longitudinal associations with stroke recurrence. Finally, we acknowledge that our findings may not generalize to reperfusion-treated or highly disabled populations. Future studies could stratify analyses by treatment status or disability severity.

## Conclusion

The combination of an elevated TyG index and ICAS burden effectively stratifies the short-term risk of stroke recurrence, with these markers showing superior predictive value when used together rather than individually. This combined approach may help to identify patients at highest risk of recurrence, potentially guiding more targeted interventions and improving patient outcomes. However, further research is needed to fully elucidate the underlying mechanisms of this association and to validate these findings in different populations.

## Data Availability

The original contributions presented in the study are included in the article/supplementary material, further inquiries can be directed to the corresponding authors.

## Glossary

BP: Blood pressure
BMI: Body mass index
CI: Confidence interval
CTA: Computed tomography angiography
MRA: Magnetic resonance angiography
DSA: Digital subtraction angiography
ECAS: Extracranial arterial stenosis
FPG: Fasting plasma glucose
HDL: High-density lipoprotein
ICAS: Intracranial arterial stenosis
IS: Ischemic stroke
IDI: Integrated discrimination improvement
LDL: Low-density lipoprotein
NIHSS: National Institutes of Health Stroke Scale
NRI: Net reclassification index
HR: Hazard ratio
TIA: Transient ischemic attack
TyG: Triglyceride-glucose index
TG: Triglyceride
WBC: White blood cell
WASID: Warfarin and Aspirin for Symptomatic Intracranial Arterial Stenosis
