# Supplementary material for: Unveiling the dual threat: combined elevated triglyceride-glucose index and intracranial arterial stenosis burden for enhanced stroke risk stratification
Source: Front Neurol. 2025 Jun 30;16:1561329. doi: 10.3389/fneur.2025.1561329 (PMC12256768; doi:10.3389/fneur.2025.1561329)
Supplement: Supplementary file 1 [file Supplementary_file_1.docx]

**Supplementary Table. Variance Inflation Factors and Tolerance Values for Model Variables**

| **Variance Inflation Factor and Tolerance** | | | | | |
| --- | --- | --- | --- | --- | --- |
| **Term** | **VIF** | **VIF**  **（CI 95%）** | **SE factor** | **Tolerance** | **Tolerance**  **（CI 95%）** |
| Age | 1.170121 | 1.096897  1.298679 | 1.081721 | 0.8546128 | 0.7700134  0.9116628 |
| BMI | 1.160469 | 1.089120  1.288941 | 1.077251 | 0.8617205 | 0.7758308  0.9181726 |
| Baseline SBP | 2.011818 | 1.817296  2.252636 | 1.418386 | 0.4970629 | 0.4439243  0.5502680 |
| Baseline DBP | 2.067674 | 1.865606  2.316913 | 1.437941 | 0.4836352 | 0.4316087  0.5360189 |
| Current cigarette smoking | 1.180186 | 1.105066  1.309015 | 1.086364 | 0.8473242 | 0.7639332  0.9049235 |
| Current alcohol drinking | 1.177361 | 1.102768  1.306098 | 1.085063 | 0.8493571 | 0.7656396  0.9068093 |
| Hypertension | 1.050803 | 1.010791  1.239169 | 1.025087 | 0.9516531 | 0.8069924  0.9893239 |
| Diabetes | 1.073251 | 1.023942  1.224110 | 1.035978 | 0.9317486 | 0.8169204  0.9766176 |
| stroke | 1.062999 | 1.017569  1.225898 | 1.031019 | 0.9407345 | 0.8157286  0.9827339 |
| Atrialfibrillation | 1.108195 | 1.048458  1.241572 | 1.052709 | 0.9023681 | 0.8054306  0.9537812 |
| Coronary heart disease | 1.082985 | 1.030412  1.226444 | 1.040666 | 0.9233735 | 0.8153656  0.9704858 |
| Antiplatelet therapy | 1.840491 | 1.669205  2.055619 | 1.356647 | 0.5433332 | 0.4864716  0.5990874 |
| Statin therapy | 1.044207 | 1.007613  1.256707 | 1.021864 | 0.9576647 | 0.7957304  0.9924448 |
| HDL | 1.136418 | 1.070043  1.265692 | 1.066029 | 0.8799582 | 0.7900815  0.9345422 |
| LDL | 1.129314 | 1.064512  1.259208 | 1.062692 | 0.8854934 | 0.7941500  0.9393973 |
| Admission NIHSS score | 1.913376 | 1.732186  2.139403 | 1.383248 | 0.5226365 | 0.4674201  0.5773052 |
